# An Adaptive Multi-Scale Network Based on Depth Information for Crowd Counting †

**DOI:** 10.3390/s23187805

**Published:** 2023-09-11

**Authors:** Peng Zhang, Weimin Lei, Xinlei Zhao, Lijia Dong, Zhaonan Lin

**Affiliations:** 1 School of Computer Science and Engineering, Northeastern University, Shenyang 110167, China; 1910609@stu.neu.edu.cn (P.Z.); 1910692@stu.neu.edu.cn (Z.L.); 2 Artificial Intelligence Research Institute Shenyang, 213 Electronic Technology Co., Ltd., Shenyang 110023, China; zhaoxinlei@china213.net (X.Z.); donglijia@china213.net (L.D.)

**Keywords:** object counting, crowd counting, deep learning, CNN

## Abstract

Crowd counting, as a basic computer vision task, plays an important role in many fields such as video surveillance, accident prediction, public security, and intelligent transportation. At present, crowd counting tasks face various challenges. Firstly, due to the diversity of crowd distribution and increasing population density, there is a phenomenon of large-scale crowd aggregation in public places, sports stadiums, and stations, resulting in very serious occlusion. Secondly, when annotating large-scale datasets, positioning errors can also easily affect training results. In addition, the size of human head targets in dense images is not consistent, making it difficult to identify both near and far targets using only one network simultaneously. The existing crowd counting methods mainly use density plot regression methods. However, this framework does not distinguish the features between distant and near targets and cannot adaptively respond to scale changes. Therefore, the detection performance in areas with sparse population distribution is not good. To solve such problems, we propose an adaptive multi-scale far and near distance network based on the convolutional neural network (CNN) framework for counting dense populations and achieving a good balance between accuracy, inference speed, and performance. However, on the feature level, in order to enable the model to distinguish the differences between near and far features, we use stacked convolution layers to deepen the depth of the network, allocate different receptive fields according to the distance between the target and the camera, and fuse the features between nearby targets to enhance the feature extraction ability of pedestrians under nearby targets. Secondly, depth information is used to distinguish distant and near targets of different scales and the original image is cut into four different patches to perform pixel-level adaptive modeling on the population. In addition, we add density normalized average precision (nAP) indicators to analyze the accuracy of our method in spatial positioning. This paper validates the effectiveness of NF-Net on three challenging benchmarks in Shanghai Tech Part A and B, UCF_ CC_50, and UCF-QNRF datasets. Compared with SOTA, it has more significant performance in various scenarios. In the UCF-QNRF dataset, it is further validated that our method effectively solves the interference of complex backgrounds.

## 1. Introduction

The crowd counting task is widely used in fields such as public transportation [1,2], bioscience [3,4], anomaly detection [5], and video surveillance [6,7]. With the continuous increase in population and the acceleration of urbanization, research on crowd counting has become a new hotspot and a challenge in the field of computer vision. In recent years, deep learning-based methods have made significant progress in the field of computer vision and CNN-based methods [8] have strong abilities in image feature representation. In 2010, Lempitsky et al. [9] first proposed the concept of the density map, which calculates the number of people per pixel by learning the mapping between local features of an image and its corresponding density map, resulting in adding spatial information of the original network. Later methods of crowd counting research have all been improved based on this method. The density map estimation-based method allows the model to directly learn the mapping relationship from pixel features to the target density distribution map, which includes density information and spatial information, and, by integrating the density map, the number of population targets can be obtained. The density map estimation-based method has good detection performance in high-density populations and has gradually become a popular method. However, the density map estimation-based method is not robust to scale changes when facing multi-scale tasks; moreover, such methods cannot obtain the most accurate predicted density map under strong background noise.

At present, the crowd counting task faces many challenges, such as changes in density and distribution in different scenes and inconsistent density distribution in local areas of the same scene. Secondly, most mainstream crowd counting algorithms are designed for specific densities or scenarios, making it difficult to fully utilize density information. So, the crowd counting task needs to handle issues such as multi-scale changes, occlusion, distorted perspective, and large-scale changes in crowd targets.

**Figure 1:**
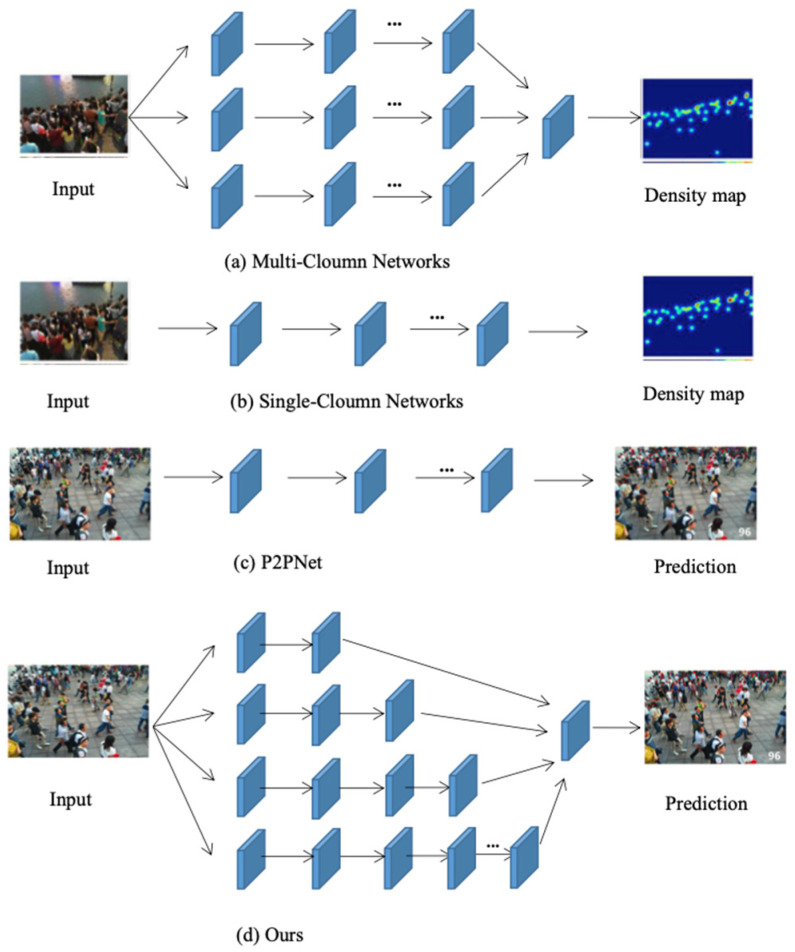
Different network structures. The blue box represents the convolutional layer. The sub-graph (**a**) shows a multi-column CNN architecture, (**b**) shows a single-column architecture, (**c**) predicts the number of people directly between P2PNet points, and (**d**) shows our approach.

The common approach adopted by researchers to address the above issues is to change the structure of the network. Reference [10] introduces a method of changing the network structure by adding different columns that can capture more multi-scale feature information and different receptive fields. As shown in Figure 1a, the first method is a multi-column neural network with different convolutional sub-networks, abbreviated as the multi-column architecture. Zhang et al. proposed the MCNN [11] network, which generates density maps through annotation files of the center pixel coordinates of all heads in the image, achieving better results than traditional manually generated density maps. There are many other methods based on multi-column network architecture, such as switch-CNN [12], CMTL [13], Hydra-CNN [14], CP-CNN [15], and SAA-Net [16]. The multi-column architecture integrates convolutional kernels of various sizes into different branches. To extract different scale features, different branches accommodate different receptive fields. Finally, the features extracted from different branches are fused to generate the final density map. Although the multi-column network architecture solves the problem of scale changes, it still cannot adapt to the interference of complex scenes. It is difficult to train networks with multiple network architectures simultaneously, because the diversity of scales that can be accommodated is limited by the number of branches. Setting the same structure for different branches can also cause a lot of information redundancy.

As shown in Figure 1b, the second method is a single-column architecture, which learns multi-scale feature information from images through different single-column neural networks by deploying a single and deeper CNN structure based on the single-column architecture, reducing the network complexity by removing the full connection layer in the network, and designing a complex convolution layer to increase the receptive field of the model. CSRNet [17] adopts a framework based on single-column architecture and introduces the concept of dilation convolution into crowd counting tasks. Cavity convolution uses a sparse convolution core to realize alternate convolution and pooling operations, which can improve the receptive field without sacrificing the cost of network parameters. ADSCNet [18] proposes an adaptive dilation convolution based on CSRNet, which can learn a dynamic and continuous dilation rate for each position and different positions use different dilation rates to adapt to scale changes. In addition, there are some methods where ADcrowdnet [19] introduces deformable convolutional layers to capture more spatial information. HA-CNN [20] introduces an attention mechanism to enhance the learning ability of the network and designs a spatial attention module to learn the spatial position of low-level features and another global attention module to learn feature maps at channel latitude. By utilizing the feature map scales of different layers in VGG16 [21], the structure is designed to enable the network to process multi-scale information. Compared with multi-column architecture (a), single-column architecture is simple and easy to train. However, the CNN method based on single-column architecture cannot effectively analyze the perspective information of any scene and its processing efficiency for scale changes is very low.

As shown in Figure 1a,b, adapting to multi-scale changes by changing the network structure (multi-column, single-column) and then generating density maps for density estimation and population counting. By integrating the density map, the total number of people can be obtained. However, for large and dense crowds in crowded areas, density map-based methods cannot provide accurate location information of individuals in the crowd.

As shown in Figure 1c, P2PNet [22] proposes a completely point-based framework (Point to Point Network) to directly predict the position of the head center point in the population, while achieving individual population localization and population counting tasks. P2PNet has excellent performance in global contexts and the individual location information provided by this framework at the point level is the foundation of downstream high-order crowd analysis tasks. P2PNet provides spatial information of character targets at various positions in the image. This helps with motion analysis for dense crowd counting tasks. In addition, the framework effectively avoids a large number of label annotations and the recognition accuracy is not affected by inaccurate pseudo boxes.

However, the accuracy of counting is very low when identifying nearby targets (where the distance between the person and the camera is relatively close), because the feature information between nearby and distant targets from the same perspective is not distinguished. The method proposed in this paper optimizes and improves the P2PNet in (c) and the multi-column network in (a). Therefore, we propose an adaptive near–far network NF-Net, as shown in (d) in Figure 1. The multi-column network in (a) extracts multi-scale features by expanding the types and sizes of network convolutional kernels, while our proposed method achieves a stepped convolutional network structure by gradually stacking network layers. Firstly, the original image is divided into different patches (this method verifies that when the original image is divided into four parts, the detection performance is best). Each patch corresponds to a network with different layers. In this way, the distant target can be assigned a smaller receptive field and the near target can be assigned a larger receptive field. Finally, the number of people in each patch is predicted and summed up to obtain the final result.

**Figure 2:**
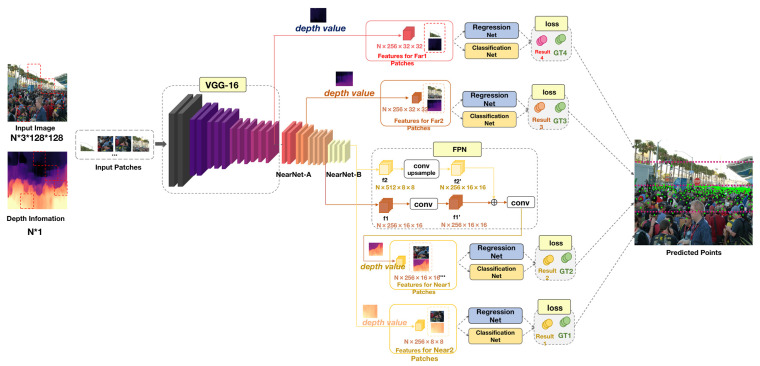
The overall framework of the proposed NF-Net. The backbone of NF-Net adopts all convolutional layers in VGG 16 [21] and a NearNet network is added later. Randomly divide the input image into four squares according to the patch, mark them with a red dashed box in the input image, and correspond to the depth information one by one. The four input patches obtained through segmentation are sequentially input into VGG16. Feature fusion layer: convolutional blocks are simplified as Conv in the graph (Section 3.1.5). By introducing depth information to reflect the density of the original image, represented by a heat map, the depth information module is described in Section 3.2. After obtaining these results, the counts of the four patches are combined to output the results.

**Figure 3:**
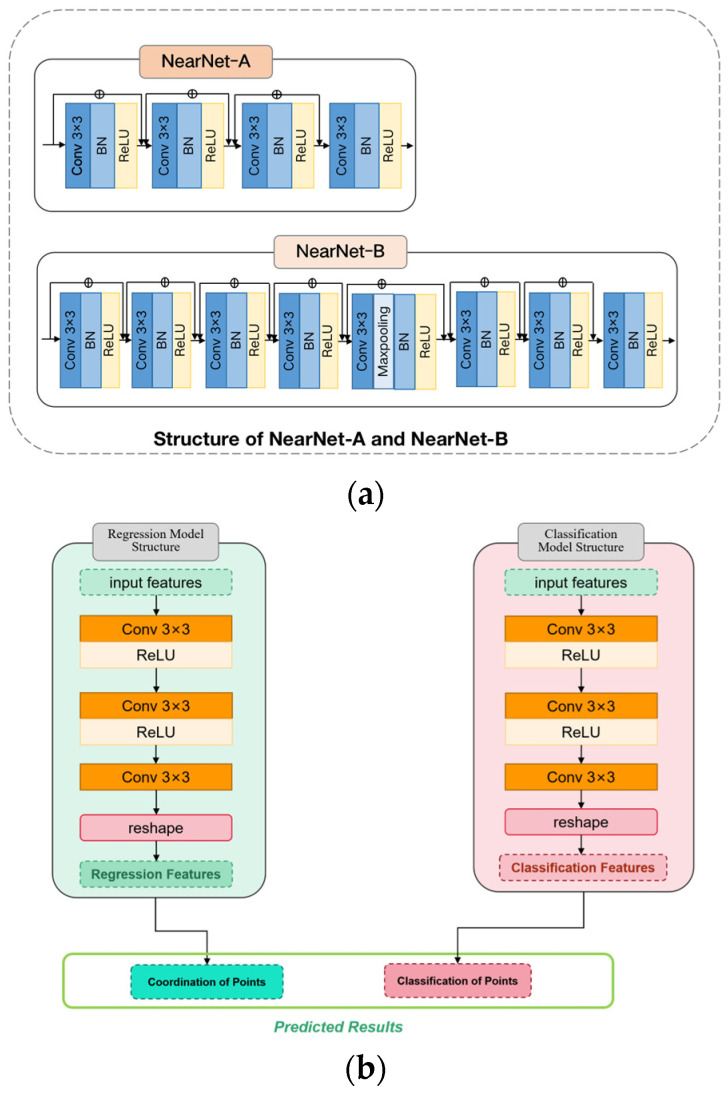
The network structure diagrams of NearNet-A, NearNet-B, and head networks. (**a**) NearNet-A and NearNet-B; (**b**) head networks.

This paper improves on P2PNet by introducing depth information weight parameters to reflect the distance of the target by mainly solving the problem of large feature information and low recognition accuracy of nearby targets. The overall framework of the proposed NF-Net is shown in Figure 2. Our main contributions are summarized as follows:

- We introduce a simple end-to-end crowd counting network, abbreviated as NF-Net. We have improved the structure of the original feature extraction network VGG16 to better focus on the differences in local feature information by deepening the layers of the feature extraction network. In order to allocate different receptive fields to populations of different densities, we propose NearNet to allocate the network to different depths. NearNet-A continuously stacks four convolutional layers, while NearNet-B continuously stacks eight convolutional layers. The network structure diagrams of NearNet-A, NearNet-B, and the head network are shown in Figure 3. The more layers stacked, the greater the receptive field that needs to be allocated to the population. Realizing distinguishing features between distant and nearby targets further improves the accuracy and robustness of the crowd counting model.
- We have designed an adaptive distance adjustment module based on depth information. By introducing depth information weight parameters to represent the distance between people and cameras, different densities of people are divided according to their density based on the distance. The fusion between features at different distances will guide the model to explicitly extract features from multiple receptive fields and learn the importance of each feature at each image location. In other words, our method adaptively encodes the size of the receptive field required for population density.
- The design of the patch selection module introduces the idea of local information. Specifically, the original image is evenly divided into four different patches, each corresponding to a different scale of population. Based on the angle taken by the camera, it can be inferred that the closer the area to the camera is, the denser the crowd is. Therefore, the patches on the top two parts of the image are divided into dense and sub-dense from top to bottom, while the bottom two parts are divided into sub-sparse and sparse from top to bottom. Finally, four different levels of crowd distribution information are formed to achieve spatial differentiation of crowds and effectively improve the recognition of nearby targets, while avoiding interference from complex backgrounds.
- Experimental evidence shows that NF-Net is effective. NF-Net has achieved state-of-the-art performance in the Shanghai Tech Part A and B, UCF_CC_ 50, and UCF-QNRF datasets. Our method surpasses the state-of-the-art P2PNet and significantly reduces MAE and RMSE. We have conducted ablation experiments and comparative analysis to verify the effectiveness of our method on the crowd counting benchmark.

We note that the shorter conference version of this paper appears in reference (Zhang and Lei) [23] and the initial conference version is only initially validated in a single dataset without a detailed analysis of the role of the patch selection module (PSM) and the deep information module (DIM). This manuscript provides more experimental data and adds density normalized average precision (nAP) to verify the robustness of the method proposed in this paper.

## 2. Materials and Methods

### 2.1. Detection-Based Methods

The detection-based method mainly detects individuals in each frame of the video and counts the number of people. Most early methods for crowd counting are based on pedestrian detection [24,25], which achieve crowd counting by detecting or segmenting individual pedestrian targets in the scene. The method proposed by Leibe et al. [26] combines local and global clues through top-down probability segmentation. However, good counting results can only be obtained in sparse scenes, so detection-based methods are only suitable for low density populations. When the crowd density increases or more complex scenarios are encountered, there is a problem of severe overlap between instances. This may lead to the prediction box being erroneously suppressed by NMS. Moreover, highly overlapping targets may have very similar characteristics. Therefore, it is difficult for detectors to generate differentiated predictions for each proposal separately. At this point, the accuracy of the crowd counting algorithm based on detection significantly decreases, resulting in the inability to obtain accurate counting results [27,28,29]. By detecting parts of the body structure, such as the head, shoulders, upper body, etc., this local detection method is easier to distinguish than whole body-based detection features and has a slight improvement in recognition performance.

### 2.2. Regression-Based Methods

The regression-based methods establish a mapping between features and the number of people to perform crowd counting. In 2015, Liu et al. [30] proposed a regression model to directly learn global counting. The model directly learns the mapping relationship from image features to counting. Chen et al. [31] proposed to transform low-level image features into a cumulative attribute space, where each dimension has a clearly defined semantic explanation that can capture how the population count values continuously accumulate and change. Idress et al. [32] proposed the method of using Fourier transform and scale invariant feature transform (SIFT) to extract features, using Markov random fields (MRF) to establish regression models, finally achieving dense crowd counting tasks. The regression-based methods rely on manually designed features such as SIFT, LBP, etc., and do not fundamentally solve the problem of crowd congestion. The regression-based method does not include spatial annotation and can only reflect the number of people but cannot locate the specific location of each target, so the overall performance is relatively mediocre.

### 2.3. Density Map-Based Methods

The latest research methods are based on density map estimation for crowd counting. The density map-based methods [33,34,35,36,37,38,39,40,41,42] generate a density map based on the annotation of the center point of the head. By integrating the density map, the total number of people can be obtained. These methods attempt to solve the problem of scale change by combining scale dependent features from multiple branches. Cao et al. [43] proposed a scale aggregation network (SANet), which borrows the architecture idea of inception and encodes it by stacking four convolutional check networks with different sizes in parallel. Decoding is performed using deconvolution layers to generate high-resolution density maps. RAZNet [44] proposed a cyclic attention scaling network to improve localization resolution and proposed a local self-attention module and a global self-attention module to simultaneously obtain local and global features. TEDNet [45] proposed a grid encoder decoder network architecture that integrates multiple decoding paths to capture multi-scale features and utilizes dense skip connections to obtain supervised information.

In addition, there are also some methods that demonstrate better performance by combining crowd counting with other tasks such as classification, detection, segmentation, etc. CFF [46] not only generates density maps through point supervision, but also generates supervised focus from segmentation and focus from global density. The focus guides the counting network from segmentation to focus on areas of interest. BL [47] proposed a new loss function, Bayesian loss, which constructs a statistical model of density contribution from the perspective of point annotation. The proposed training loss does not constrain the values of each pixel in the density map, but rather provides more reliable supervision of the counting expectation of each annotation point. DM-count [48] uses optimal transport to measure the similarity between predicted density maps and ground truth maps.

### 2.4. Transformer-Based Methods

Due to the widespread application of vision transformer (ViT) [49] in computer vision tasks, many crowd counting tasks [50,51] currently use transformer-based methods. The transformer-based method uses an encoder–decoder architecture with a self-attention mechanism for feature extraction, making the model more focused on global information and achieving direct prediction of the number of people in the entire image. In 2022, Liang et al. [52] first applied the transformer method to crowd counting tasks. TransCrowd segments the input image into fixed size blocks, each of which is linearly embedded. The feature embedding sequence is inserted into the transformer encoder and finally generates the predicted number of people. CCTrans [53] uses the same infrastructure as TransCrowd, but the backbone of the feature encoder uses Twins [54], which is more powerful than ViT. Crowdformer [55] introduces a pyramid-structured transformer to extract multi-scale features with global backgrounds. The backbone network adopts PVTv2 and proposes a feature aggregation module to fuse features from different stages of transformers. Finally, the number of people is estimated based on regression heads.

Compared with mainstream CNN-based methods, although this type of method can save data annotation costs, it has a large number of model training parameters and is not suitable for densely populated scenarios. In addition, transformer-based methods focus more on global information. Due to the small size of crowd targets, many texture details will gradually be lost during the continuous iteration of the network. The attention mechanism of the transformer focuses more on the global field of view and is not good at detecting small targets with local high density.

## 3. Methodology

The basic idea of our method is to introduce deep information to improve and solve the problem of poor recognition ability in sparse scenes in crowd counting, thereby improving the flexibility and ability of the overall model. For this purpose, we have designed a framework for a near far adaptive network (NF-Net), as shown in Figure 2. The overall framework of NF-Net proposed in this article consists of six parts: backbone, regression branch, classification branch, feature fusion, depth information module, and patch selection module.

The basic structure of the near and far network is introduced in Section 3.1. The backbone (Section 3.1.1) is composed of VGG16 and NearNet networks (Section 3.1.4). The NearNet network is a network with multiple layers of convolutional stacking and head branch composed of regression head (Section 3.1.2) and classification head (Section 3.1.3). For the depth information module, we have introduced depth information to reflect the density of different populations (Section 3.2). The patch selection module is divided into four patches to achieve spatial division of the population and optimize through loss (Section 3.3). In addition, we describe the calculation process of the loss function.

### 3.1. Near and Far Network

#### 3.1.1. Backbone

The VGG16 neural network model uses 3 × 3 convolution and 2 × 2 pooling from beginning to end, making the model design simple. Therefore, we use the first 13 convolutional layers in VGG 16-BN to extract deep features. For the VGG16 convolutional neural network, its 13 layers of convolution and 5 layers of pooling are responsible for feature extraction; the final 3 layers of the full connection layer are responsible for completing the classification task. NF-Net is based on the VGG16 model and adds NearNet-A and NearNet-B modules after the VGG16 network. The network structure of the NearNet-A and NearNet-B modules is shown in Figure 3a.

#### 3.1.2. Regression Head

As shown in Figure 3b, the left side shows the network structure diagram of the regression head. Two branches are used to simultaneously predict a set of point coordinates, where there is a one-to-one matching relationship between the point proposal sets and the ground truth point sets. For regression branches, due to the inherent translation invariance of the convolutional layer, it needs to predict the offset of point coordinates. Finally, the coordinate set of the predicted points are output.

#### 3.1.3. Classification Head

As shown in Figure 3b, the right side shows the network structure diagram of classification head. For the classification branch, by judging the pedestrian targets at each position in the feature map, the foreground and background can be distinguished. The classification branch outputs the confidence score $\left\{C_{j}|i\in \left\{1,\ldots ,M\right\}\right\}\;$ corresponding to the predicted point set through softmax normalization.

#### 3.1.4. NearNet

The deeper the network, the larger the receptive field of the model. In order to allocate different receptive fields to populations with different densities, we propose NearNet-A and NearNet-B to allocate networks to different depths. Among them, NearNet-A continuously stacks 4 convolutional layers and NearNet-B continuously stacks 8 convolutional layers. The purpose is to allocate small receptive fields to high-density populations, while low-density populations are allocated larger receptive fields.

**Figure 4:**
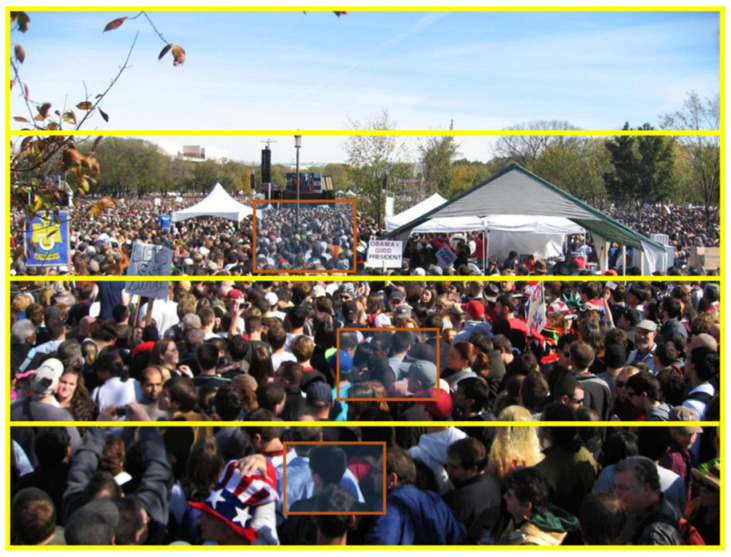
The schematic diagram of dividing the patches. The three red boxes respectively display the number of local heads, and the number of people in the red boxes gradually decreases from far to near.

We divide the density of the population into four types from top to bottom, namely dense, sub-dense, sub-sparse, and sparse. From Figure 4, it can be seen that the image is divided into four parts by a yellow line, with each part having the same size of red box. However, the number of human heads does indeed decrease due to the obvious depth information in the image. The head in the distance is small and the number of people in the red box is significantly higher than in the bottom red box. Reflected in the 2D image, it indicates that the red boxes in the distance are denser, while the red boxes in the vicinity are sparse. Because the distance between the person and the camera in each picture is from far to near, the image can be divided into far patches and near patches. Among them, far patches include Patch A (farthest) and Patch B (second farthest); near patches include Patch C (second nearest) and Patch D (nearest). The network layer is kept unchanged for the farthest target (Patch A) and the original settings of P2PNet are maintained. The purpose of this setting is to increase the depth of the network, further enhance the ability to extract features near, achieve ladder growth in the number of network layers of distant and near targets, and achieve the goal of allocating different receptive fields to targets with different distances; furthermore, the accuracy for nearby targets is improved.

#### 3.1.5. Feature Fusion Layer

The feature fusion part is improved on the basis of P2PNet, which does not adopt any multi-scale feature fusion strategy. We use FPN feature pyramids to fuse various patches. Upsampling paths are introduced to obtain fine-grained depth feature maps. Horizontal connections are used to fuse the upsampled high semantic features with shallow localization details. Firstly, for the distant targets of Patch A and Patch B, the model does not perform feature fusion. When training the model for the nearby target Patch C, it is weighted and fused with the features of Patch D before outputting. As the distance between the crowd continues to come closer, the feature information of the target will also become more prominent. Patch D represents the nearest feature, with the most clear and intuitive feature information. Some features between Patch C and Patch D are similar; therefore, learning the feature information of the nearest target in Patch D can help improve the local feature extraction ability in Patch C.

### 3.2. Depth Information Module

**Figure 5:**
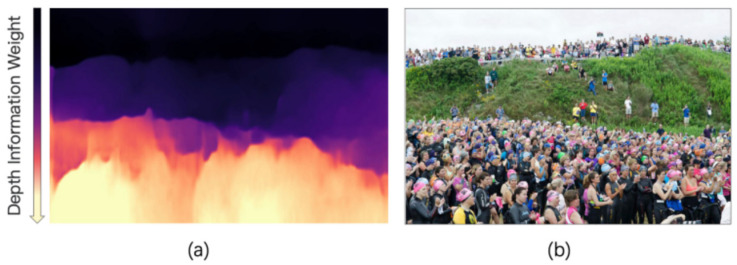
The illustration of depth information module [23]. (**a**) represents a visual heatmap of the depth information of the image, while (**b**) represents the original image.

We propose a depth module based on depth information weight that can automatically adjust distance (DIM). Figure 5a reflects the heat map of the distance and proximity of the population. The depth information weight gradually decreases from top to bottom and the color also gradually becomes lighter. The lighter the heat map color, the more obvious the feature information, which is used to determine the proximity of the target (whether it is a large or small target), with the aim of adapting to different sizes of people based on proximity.

Before the predicted image is fed into the model, we calculate the depth values of different patches and normalize them to the 0–1 range. The obtained depth values represent the distance of the current patch and are also fed into the network structure as auxiliary parameters. When training a network, the patch selects which network structure to enter based on its corresponding auxiliary parameters, that is, if the depth information is large, it is sent to the farthest network branch (i.e., the network branch for training smaller targets) and if the depth information is small, it is sent to the nearest network branch (i.e., the network branch for training larger targets). By feeding appropriate data into the appropriate network structure in a targeted manner, deep information can assist in updating and learning network parameters.

Figure 5b represents the original image. It can be seen that, along the direction from far to near, there are significant differences between the features of the distant (upper) target and the features of the nearby (lower) target. There is more occlusion between distant targets and the proportion of people is small, so a smaller receptive field is needed. The near target is affected by the shooting angle. The near target has a larger proportion of heads and a sparse population distribution, so a larger receptive field is needed. This paper proposes that the weight of depth information is calculated by the following equation:

(1) $$D_{W_{AB}}=\frac{\sum_{i=l,j=h}^{K}W_{AB}\left(x_{i},\;y_{i}\right)}{\sum_{i=1,j=1}^{C}W_{AB}\left(x_{i},\;y_{i}\right)}$$

where l and h are the starting abscissa and ordinate of the proposal point, K is the ending abscissa and ordinate of the proposal point, and C is the number of total headcounts. Therefore, $D_{W_{AB}}$ is the weighted average of the normalized picture description.

When P2PNet samples data, a “random sampling” method is used (randomly selecting areas within the image for sampling). Due to the fact that the camera for dense crowd statistics still moves from far to near, it is highly likely that during the sampling process, the head of the farthest target only has a few pixels, while the contour of the head can be clearly seen in the near area (i.e., the features of the distant and near targets are not the same), as shown in Figure 4. The random approach results in different sizes of people being unable to train the network in a targeted manner, which is also one of the reasons why existing crowd counting schemes cannot effectively identify nearby heads. So, we can use “branch sampling” to solve the lack of sample scale diversity to a certain extent, that is, DIM uses the quartile method for branch sampling, which can effectively fully sample small distant targets and large nearby targets. However, due to different target features, branch sampling requires corresponding network models. The introduction of depth information provides the near–far relationship of the samples to be trained. Based on “branch sampling”, the degree of near–far of the samples is more accurately and directly divided through the extraction and statistics of depth values, enabling data to be more targeted and fed into different structures of far/near sub-networks. This strategy improves the feature scale and representation in the network while increasing sample diversity and enhances the counting effect of larger heads in close proximity.

### 3.3. Patch Selection Module

**Figure 6:**
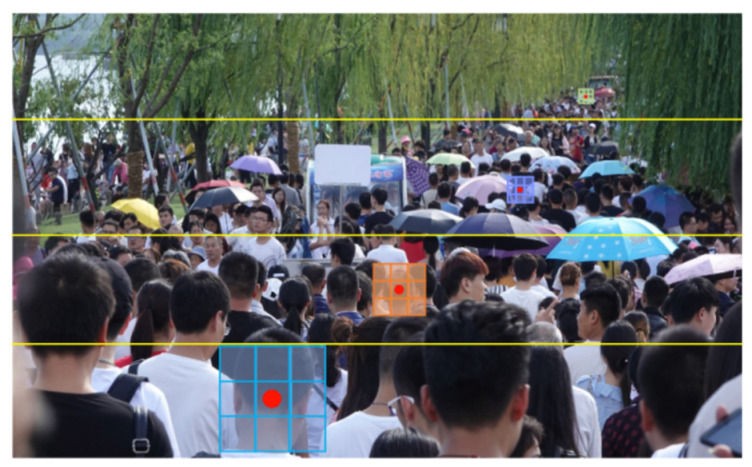
The illustration of the patch selection module [23]. The image is divided into four patches, with red dots representing the center pixel of the head. Among the four different patches, there are four convolutional blocks of different colors (green, purple, orange, and blue in order); It can be seen that the convolutional blocks at the center of the head gradually increase in size from top to bottom, and the feature information of the head also gradually becomes clear.

The patch selection module adopts a quartile strategy. Quartile, also known as quartile, refers to the arrangement of all values from small to large in statistics, dividing them into four equal parts and finally obtaining the values at the three dividing points. Due to the overall architecture of NF-Net being divided into four sub-networks, the samples to be trained in the input network should be divided into four levels. Firstly, during the data-loading process, we obtain the depth values of each patch and then obtain the depth quartiles from the set of depth values of several patches under a single image to define the depth range of specific training data. Secondly, based on the depth value of each patch and the calculated quartile, several patches are “divided” into different levels to obtain the “far near index” (the index values are 0, 1, 2, and 3, representing four sub-networks). Finally, during model training, each patch selects the network scale based on its “far near index” (Figure 6).

After feature fusion, the target’s proximity is determined based on the depth information weight and the image is evenly segmented. The specific implementation steps are as follows:

1. First, divide the original image into four patches according to coordinates;
2. Second, calculate the maximum value $D_{max}$ and minimum value $D_{min}$ of each patch according to the Formula (1) $D_{W_{AB}}$;
3. Third, when $D_{crip}>\mu$ starts to split the far and near branches, the formula for the division factor $D_{crip}$ is as follows:
  (2) $$D_{crip}=D_{max}-D_{min}$$
4. We first set τ to 2, 4, and 8 scenarios and experimental comparison is conducted on the UCF_CC_50 dataset. The results show that, when τ = 4, μ = 0.15, the MAE value is the smallest and the accuracy of the model reaches its best.

### 3.4. Loss Design

The patch selection module divides the original image into four patches and the feature information corresponding to the four patches is different. Therefore, in order to better train NF-Net, we calculate the respective losses for each of the four patches. By fine-tuning the parameters in the four patches, we update the network based on their respective loss functions and further optimize the network. After the ground truth is obtained, we compute the Euclidean loss $L_{loc}$ to supervise the point regression branch and use the cross-entropy loss $L_{cls}$ to train the classification head. The final loss $L_{\left(0,1,2,...,\tau -1\right)}$ function is the sum of the above two losses, where τ indicates the number of split patches. The loss calculation formula is as follows:

(3) $$L_{cls}=-\frac{1}{M}\left\{\sum_{i=1}^{N}log{C^{\prime }}_{\xi (i)}+\lambda_{1}\sum_{i=N+1}^{M}log\left(1-{C^{\prime }}_{\xi \left(i\right)}\right)\right\}$$

(4) $$L_{loc}=\frac{1}{N}\sum_{i=1}^{N}{\left\|p_{i}-{p^{\prime }}_{\xi \left(i\right)}\right\|}_{2}^{2}$$

(5) $$L_{\left(0,1,2,...,\tau -1\right)}=L_{cls}+\lambda_{2}L_{loc}$$

where $p_{i}$ is the ground truth point and ${p^{’}}_{\xi \left(i\right)}$ is the proposal point. When $\;i\in \left\{1,\ldots ,N\right\}$, it means that the successful matching of the two is a positive sample. When $i\in \left\{N+1,\ldots ,M\right\}$, it means that the failed matching of the two is a negative sample. ${C^{’}}_{\xi (i)}$ is the confidence score of the proposal point.$\;{\left\|\cdot \right\|}_{l_{2}}$ denotes the Euclidean distance, $\lambda_{1}$ is a reweight factor for negative proposals, and $\lambda_{2}$ is a weight term to balance the effect of the regression loss.

## 4. Experiment

All experiments are implemented by Python 1.13.0, with conduct training and testing on the server. The server is configured with an Intel (R) Core (TM) i9-9900K CPU, Ge Force RTX 3090Ti, 11GB of memory, and Nvidia GPU Compute Capacity ≤ 8.6 (Geforce RTX30). The experiment in this paper uses the same initialization parameters as P2PNet, unless otherwise specified. The input image size of the model is 128 × 128. The batch size is 16 and SGD optimization is used for 100 iterations. In order to evaluate the performance of NF-Net, we conduct experimental evaluations on three benchmark datasets: Shanghai Tech dataset [12], UCF_CC_50 [49], and UCF-QNRF [33]. These three datasets all mark large-scale populations and have clear resolutions, including outdoor, indoor, different weather, lighting, and various scenarios in different regions, which can effectively verify the effectiveness and robustness of the model. We introduce the dataset and evaluation indicators used in the experiment. The NF-Net experimental results are compared and analyzed with advanced methods. We also conduct ablation experiments to investigate the contribution of PSM to the model performance.

### 4.1. Datasets

The Shanghai Tech dataset [12] is a classic public dataset suitable for the field of dense population counting, consisting of a total of 1198 images and 330,165 annotations. According to the density of the samples, they are divided into two parts: part A (SHA) and part B (SHB). SHA’s images are randomly collected from the internet. SHA contains 482 images, with a minimum of 33 annotations per image and a maximum of 3139 annotations. The training set consists of 300 images, with the remaining 182 images forming the test. A total of 716 images of SHB are collected from bustling urban streets in Shanghai with outdoor shooting scenes. The number of labels in each image ranges from 9 to 578; 400 images are used for training and 316 images are used to test the model.

The UCF_ CC_ 50 [49] dataset is a dataset used to calculate the number of densely populated individuals in images. This dataset integrates information from three aspects and implements smoothness constraints on nearby patches to improve the estimation of incorrect patches, resulting in a better estimation of the number of people in the image. The number of images in the dataset is relatively small, only 50, but the number of people varies greatly, including a total of 63,974 annotations in the center of the head. The number of people in each image ranges from 94 to 4543. The image is collected from the US image service website FLICKR.

The UCF-QNRF [33] dataset was released by the University of Florida in 2018 and is widely used in crowd counting tasks. UCF-QNRF has a large number of pedestrian markers. There are a total of 1535 images, with over 1.25 million character annotations. Among them, there are 1201 images in the training set and 334 images in the test set. Each image has a larger resolution, reaching 2013 × 2902 pixels. Compared with other data sets, UCF-QNRF contains large-scale labeled human bodies with multiple scenes, multiple perspectives, and multiple light and density changes, so it is very suitable for training depth convolutional neural networks. In addition, it also includes real outdoor scenes from around the world, such as buildings, vegetation, sky, and roads, which is of great significance for studying the density of people in different regions.

### 4.2. Evulations

We select mean absolute error (MAE) and root-mean-square deviation (RMSE) as evaluation indicators. MAE is usually used to evaluate the accuracy of population estimation; the smaller the value, the better the accuracy of the algorithm. RMSE is usually used to measure the robustness of the algorithm; the smaller the value, the stronger the robustness of the model. The calculation formula for these two indicators is as follows:

(6) $$\;MAE=\frac{1}{N_{test}}\sum_{i=1}^{N_{test}}\left|C_{i}^{Prediction}-C_{i}^{Ground}\right|$$

(7) $$RMSE=\;\;\sqrt{\frac{1}{N_{test}}\sum_{i=1}^{N_{test}}{\left(C_{i}^{Prediction}-C_{i}^{Ground}\right)}^{2}\;}$$

where N is the number of images in the test set. $C_{i}^{Prediction}$ is the estimated value corresponding to the *i*th test image. $C_{i}^{Ground}$ is the ground truth.

**Figure 7:**
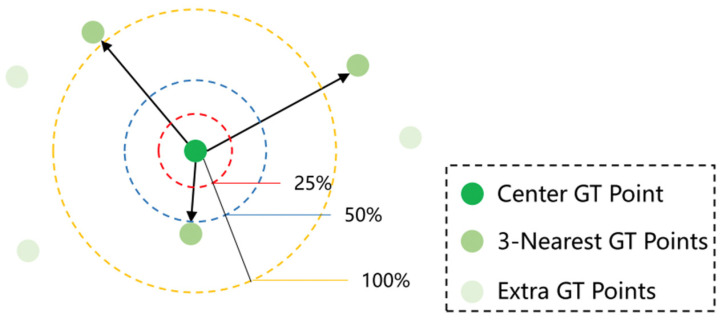
Illustration for different levels of localization accuracy in nAP [22]. The yellow circle indicates the region within $d_{kNN}\left(p_{i}\right)$ pixels from the center GT point $p_{i}$. The blue circle represents a value of 0.5 for δ, while the red circle represents a value of 0.25 for δ.

This paper also cites the density normalized average precision (nAP) (proposed in P2PNet [22] as an evaluation indicator) and evaluates the model together with MAE and RMSE. nAP can reflect the performance in the target space of the population. nAP is calculated based on average accuracy, which is the area under the precision recall (PR) curve. The PR curve can be easily obtained by accumulating binary lists. In the binary list, true positive (TP) predictions are indicated by 1; false positive (FP) predictions are indicated by 0. Specifically, given the set of all predicted head points P, we first sort the point list from high to low using their confidence scores. Then, based on predefined density perception standards, we sequentially determine whether the studied points are TP or FP. We apply sequential correlation, where the predictions with higher scores are first correlated. Therefore, TP prediction can be easily obtained through simple threshold filtering during inference. The density perception standard formula is shown in (8). The larger the value of nAP, the closer the pixel distance between the predicted point and the actual point. As shown in Figure 7, the positioning of threshold δ at different levels in nAP is explained.

(8) $$\left({\hat{p}}_{j},p_{j}\right)=\left\{\begin{array}{c} 1,\;\;\;\frac{ifd\left({\hat{p}}_{j},p_{j}\right)}{d_{kNN}\left(p_{i}\right)}<\delta ,\\ 0,\;\;\;\;\;\;otherwise,\;\;\;\;\;\;\; \end{array}\right.$$

where $d\left({\hat{p}}_{j},p_{j}\right)$ =$\;{\left\|{\hat{p}}_{j}-p_{i}\right\|}_{2}$ denotes the Euclidean distance and $d_{kNN}\left(p_{i}\right)$ denotes the average distance to the k-nearest neighbors of p_i_. We use a threshold δ to control the desired localization accuracy.

## 5. Discussion

In this section, we evaluate our method in the Shanghai Tech A&B and UCF_CC_50 datasets by comparing the counting performance of UCF-QNRF in several challenging datasets with some existing representative methods to verify the latest level of our method.

### 5.1. Comparison with State-of-the-Art Method

Shanghai Tech [12]: the comparison results in the Shanghai Tech Part A&B dataset are shown in Table 1. It can be seen that the method proposed in this paper achieves the best performance in both MAE and RMSE in the SHA dataset. The MAE mean absolute error of the method in this paper is 56.26, which is 2.54% less than the P2PNet ranking second. The RMSE root-mean-square deviation reaches 93.24%. In addition, our method also has significant advantages in RMSE, surpassing advanced algorithms such as DSSI-Net [56], SDANet [57], AMRNet [58], ASNet [59], FDC [60], and DM-Count [48] and reducing by 4.23% compared with P2PNet. In the SHB dataset, MAE and RMSE reach 6.6 and 11.0, respectively. Compared with the state-of-the-art GL [61], our method reduces its performance in MAE by 0.7%. The experimental results show that the effectiveness of the depth information module proposed in this paper reduces errors and improves accuracy.

**Figure 8:**
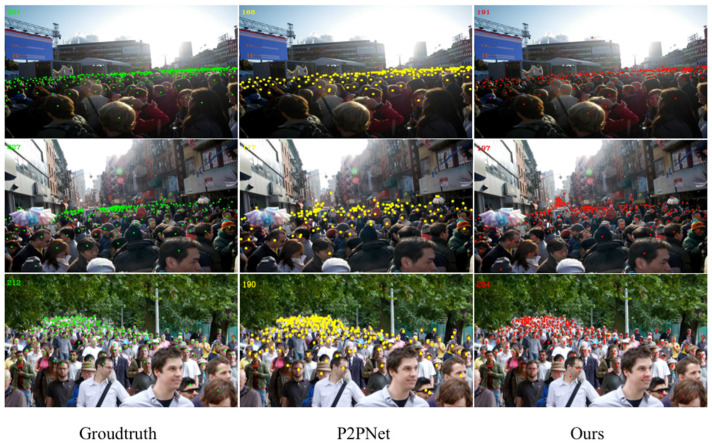
The comparison of experimental results based on Shanghai Tech Part A.

**Figure 9:**
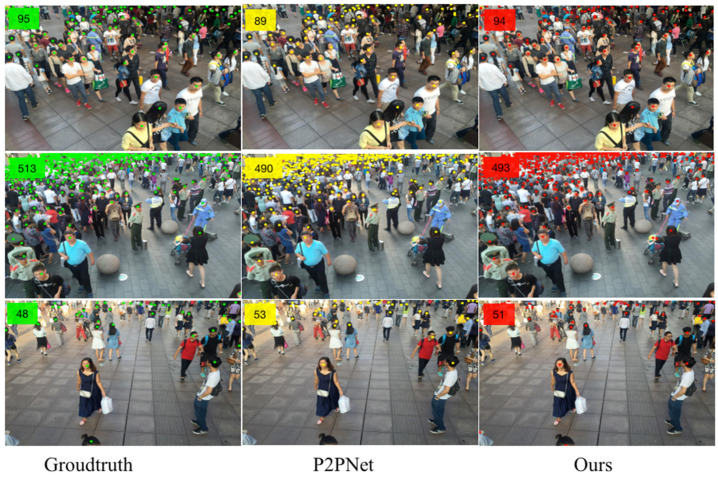
The comparison of experimental results based on Shanghai Tech Part B.

The counting effects of NF-Net in the Shanghai Tech Part A&B dataset are shown in Figure 8 and Figure 9, respectively. In order to visually demonstrate the predictive effect of the method proposed in this paper, the leftmost image is the input original image and the number of ground truth numbers is in the upper left corner of the first column. The second and third columns, respectively, compare the predicted results generated by P2PNet and our method in the same configuration environment. The predicted count results are all marked in the upper left corner of the image. In Figure 8, NF-Net has more predicted people than P2PNet, which is closer to the GT value. The Shanghai Tech dataset has a higher degree of Part A density, indicating that our method also has good recognition performance in dense populations. In Figure 9, the number of people in Part B of the Shanghai Tech dataset is relatively sparse compared with Part A. The accuracy of the comparison model between NF-Net and P2PNet has been greatly improved.

UCF_ CC_ 50 [49]: this paper mainly focuses on the accuracy of the UCF_ CC_ 50 test set compared with other models. Under the same configuration environment, the algorithm proposed in this paper is compared with other classic dense crowd counting algorithms from recent years. The experimental results are shown in Table 2, with the best results in each column highlighted in bold. The method of this paper is applied in the UCF_ CC_ 50 dataset; the mean absolute error MAE reaches 112.7 and the RMSE reaches 214.33. The results of NF-Net are significantly superior to current advanced algorithms such as Crowd-CNN [62], IG-CNN [63], D-ConvNet [64], DRSAN [65], S-DCNet [66], etc.

**Figure 10:**
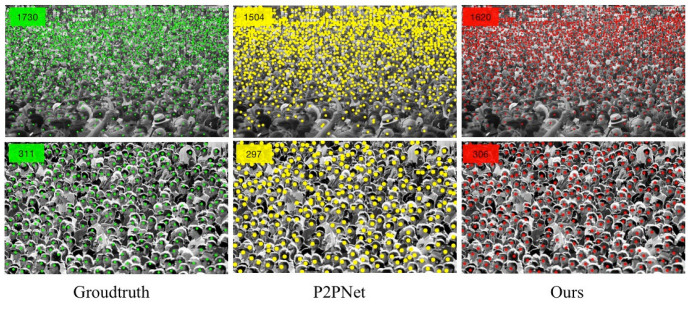
The comparison of experimental results based on UCF_CC_50.

These results show that the idea of dividing patches in the patch selection module is feasible for crowd counting tasks. Four different patches are distinguished to allocate different receptive fields, which helps the performance of the model to achieve a fundamental improvement. The detection performance of the UCF_CC_50 dataset is shown in Figure 10. The detection performance is compared with that of P2PNet. The method proposed in this paper also achieves good results when dealing with ultra-dense populations.

**Figure 11:**
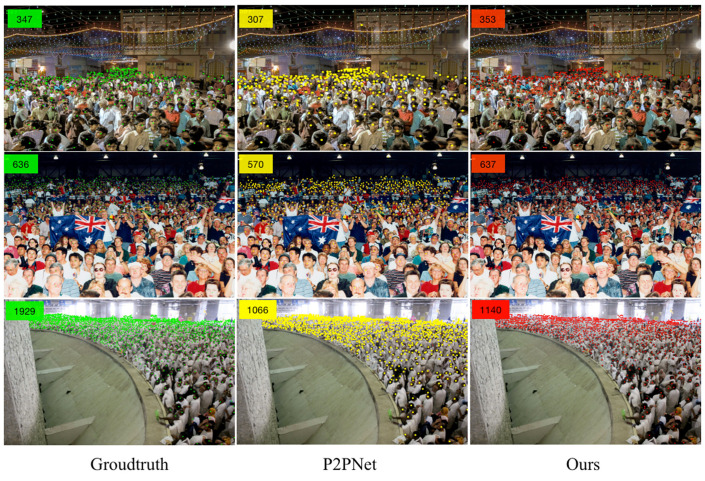
The comparison of experimental results based on UCF-QNRF.

As shown in Table 3, comparing the accuracy of the UCF-QNRF test set with other models, the MAE of NF-Net in the UCF-QNRF dataset reaches 144.06. The root-mean-square deviation RMSE also achieves good performance and the RMSE reaches 278.8 (123.59 lower than P2PNet). Because the UCF-QNRF dataset contains multi-scene and multi-angle crowd density images and, compared with other datasets, the image clarity is also higher, which helps to distinguish the density and sparsity of crowds. Therefore, in Table 4, compared with other classic dense crowd counting algorithms in recent years, NF-Net also achieves optimal performance. The detection performance of the UCF-QNRF dataset is shown in Figure 11.

Overall, our method proposed in this paper introduces the DIM module and PSM module and achieves good performance in three benchmark datasets (Shanghai Tech Part A and Part B, UCF_CC_50, and UCF-QNRF).

### 5.2. Ablation Study

Finally, ablation experiments are conducted on the test sets of Shanghai Tech Part A and Part B to confirm the benefits of the PSM module and to introduce depth information weighting. As shown in Table 4, our method divides the original image into four patches. Patches B, C, and D all reduce MAE errors; MAE decreased by 13.25, 5.59, and 1.59, respectively, compared with P2PNet. The RMSE of patches B and C also greatly reduces, with patch C and patch D generally being close to the target (i.e., the distance between the person and the camera is close, the features are large, and the head information is clear). It can be seen that the PSM module in this paper has improved performance under close targets. Due to significant differences in the experimental environment configuration between this article and P2PNet, the MAE of NF-Net in patch A reaches 32.54, slightly higher than P2PNet’s 24.50. The ablation experiment proves that the performance of the model is improved by properly distinguishing the characteristic differences between different densities and corresponding to different receptive fields.

**Figure 12:**
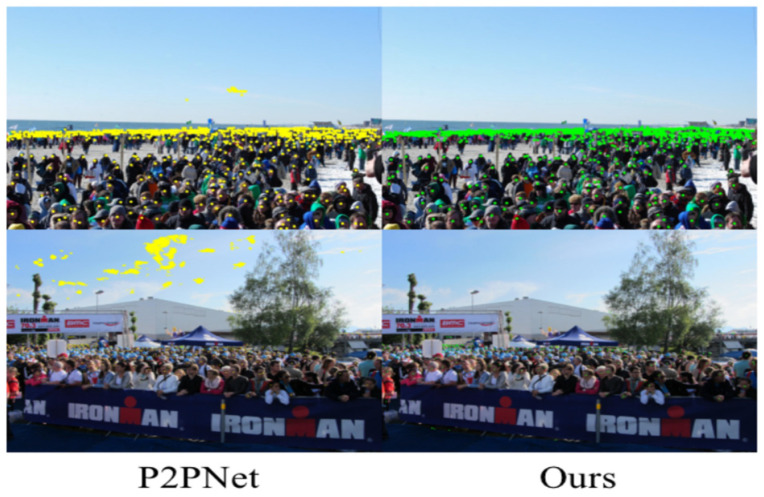
Comparison of recognition errors in the UCF-QNRF dataset.

The PSM module proposed in this paper effectively solves the important challenge of complex background interference in current crowd counting tasks. We validate the performance of the PSM module on the UCF-QNRF dataset. The PSM module distinguishes the network after the patch to avoid feature errors caused by direct input of the original image, as shown in Figure 12. P2PNet can cause large-scale false positives in some outdoor scenes. The left side of Figure 12 shows the image predicted by P2PNet, which has recognition errors in the background. This paper introduces a layered strategy to improve this problem.

As shown in Table 5, this method is applied in UCF_ CC_ 50 using nAP in UCF-QNRF to compare the performance of P2PNet to the method proposed in this paper. NAP adopts three different thresholds, δ, corresponding to the average accuracy of predicting individual points under different positioning accuracies. When δ is 0.5, it represents the evaluation within the nearby target area. In addition, when the δ value is 0.25 or 0.05, it is suitable for areas with a high concentration of targets in areas with large numbers of people far away. From Table 5, it can be seen that our method achieves better results in nAP compared with P2PNet under three different positioning accuracies. Specifically, nAP (0.50) is around 55% in all datasets, which meets many practical application needs. In the UCF_ CC_50 dataset, nAP (0.50) improves by 17.12% compared with P2PNet. The accuracy of nAP (0.05) also surpasses P2PNet, proving that the proposed method significantly improves localization performance.

## 6. Conclusions

In this paper, we demonstrate the importance of depth information in crowd counting tasks and propose an end-to-end CNN architecture, named NF-Net. (1) In order to enhance the information fusion between multiple scales, we fuse the characteristics of near targets in sparse scenes by increasing the depth of the network. For distant targets, smaller receptive fields are allocated and, for near targets, larger receptive fields are allocated. (2) We propose the depth information module (DIM), which introduces depth information weights to calculate the distance between people and the camera in order to enhance feature representation. By judging the distance between people, we achieve multi-scale feature modeling of sparse and dense populations while avoiding errors in sparse scenes. (3) We design the patch selection module (PSM), which uses a quartile strategy to learn different levels of scales and divides images into four types of patches based on distance to extract different feature information.(4) After dividing into four patches, we calculate their respective losses and predict them separately. Finally, the experimental results indicate that in the Shanghai Tech Part A&B, UCF_CC_50, and UCF-QNRF datasets, NF-Net has relatively advanced counting accuracy and robustness. The evaluation results indicate that our population counting model can robustly calculate populations and improve accuracy. NF-Net can help networks focus more on the effective feature information in images. By introducing depth information in images, it effectively solves the problem of detecting small distant targets and large nearby targets with different features in dense crowds. It can adaptively cope with multi-scale changes and provide new ideas for crowd counting tasks. Our proposed deep information module can also assist other detection tasks and improve the performance of the model. An effective dense crowd counting model has important practical significance for actual monitoring systems and effective prevention of overcrowding and stampede incidents. We hope that the work of this article can promote the widespread practical value of dense population algorithms in real-world scenarios.

## Figures and Tables

**Table 1:** The performance comparison of SHA and SHB datasets.

Methods	Venue	Shanghai Tech Part A		Shanghai Tech Part B	
		MAE	RMSE	MAE	RMSE
TEDNet [45]	CVPR2019	64.2	109.1	8.2	12.8
CAN [35]	CVPR2019	62.3	100.0	7.8	12.2
BL [47]	ICCV2019	62.8	101.8	7.7	12.7
DSSI-Net [56]	ICCV2019	60.6	96.0	6.8	10.3
SDANet [57]	AAAI2020	63.6	101.8	7.8	10.2
AMRNet [58]	ECCV2020	61.59	98.36	7.02	11.0
DM-Count [48]	NeurIPS2020	59.7	95.7	7.4	11.8
ASNet [59]	CVPR2020	57.78	90.13	-	-
FDC [60]	ICCV2021	65.4	109.2	11.4	19.1
GL [61]	CVPR2021	61.3	95.4	7.3	11.7
P2PNet [22]	CVPR2021	58.8	97.47	-	-
CCTrans [53]	CVPR2022	64.4	95.4	7.0	11.5
Crowdformer [55]	IJCAI2022	62.1	94.8	8.5	13.6
Ours	-	56.26	93.24	6.6	11.0

**Table 2:** The performance comparison of UCF_CC_50 datasets.

Methods	Venue	UCF_CC_50	
		MAE	RMSE
Crowd-CNN [62]	CVPR2015	467.0	498.5
IG-CNN [63]	CVPR2018	291.4	349.4
D-ConvNet [64]	CVPR2018	288.4	404.7
CSRNet [17]	CVPR2018	266.1	397.5
SANet [43]	ECCV2018	258.4	334.9
DRSAN [65]	IJCAI2018	219.2	250.2
CAN [35]	CVPR2019	212.2	243.7
BL [27]	ICCV2019	229.3	308.2
DSSI-Net [56]	ICCV2019	216.9	302.4
S-DCNet [66]	ICCV2019	204.2	301.3
AMRNet [58]	ECCV2020	184.0	265.8
DM-Count [48]	NeurIPS2020	211.0	291.5
ASNet [59]	CVPR2020	174.8	251.6
P2PNet [22]	CVPR2021	181.6	249.39
CCTrans [53]	CVPR2022	245.0	343.6
Crowdformer [55]	IJCAI2022	229.6	360.3
Ours	-	112.7	214.33

**Table 3:** The performance comparison of UCF-QNRF datasets.

Methods	Venue	UCF-QNRF	
		MAE	RMSE
Idrees et al. [32]	CVPR2013	315	508
MCNN [12]	CVPR2016	277	426
CMTL [14]	AVSS2017	252	514
CL [33]	ECCV2018	132	191
CSRNet [17]	CVPR2018	120.3	208.5
CAN [35]	CVPR2019	107.0	183.0
S-DCNet [66]	ICCV2019	104.4	176.1
DSSI-Net [56]	ICCV2019	99.1	159.2
BL [47]	ICCV2019	88.7	154.8
AMRNet [58]	ECCV2020	86.6	152.2
Ha-CNN [20]	IEEE TIP2020	118.1	180.4
P2PNet [22]	CVPR2021	178.78	402.39
Ours	-	104.06	178.80

**Table 4:** The ablation study of NF-Net in SHA and SHB datasets [23].

Methods	Shanghai Tech Part A		Shanghai Tech Part B	
	MAE	RMSE	MAE	RMSE
P2PNet (patch A)	24.50	49.27	-	-
P2PNet (patch B)	28.14	50.45	-	-
P2PNet (patch C)	17.34	48.69	-	-
P2PNet (patch D)	4.66	10.00	-	-
Ours (patch A)	32.54	70.11	6.17	4.0
Ours (patch B)	14.96	47.31	1.41	1.0
Ours (patch C)	11.75	38.91	0.80	2.0
Ours (patch D)	3.07	10.00	0.59	1.0

**Table 5:** Overall performance of NF-Net.

nAP	UCF_CC_50		UCF-QNRF	
	P2PNet	Ours	P2PNet	Ours
δ = 0.50	39.87%	56.99%	52.7%	54.34%
δ = 0.25	17.68%	21.60%	23.31%	24.55%
δ = 0.05	1.29%	0.92%	1.65%	2.11%

## Data Availability

Not applicable.
